# Forensic Odontology, a Boon and a Humanitarian Tool: A Literature Review

**DOI:** 10.7759/cureus.7400

**Published:** 2020-03-24

**Authors:** Roohi Afshan Kaleelullah, Pousette Hamid

**Affiliations:** 1 Dentistry, California Institute of Behavioral Neurosciences and Psychology, Fairfield, USA; 2 Neurology, California Institute of Behavioral Neurosciences and Psychology, Fairfield, USA

**Keywords:** forensic odontology, identification, bite marks, post-mortem, ante-mortem, odontology, forensic, dental age, tooth age

## Abstract

Our society faces fresh challenges in every conceivable way, from an increase in the crime rate to a rise in natural disasters. In spite of the leaps in modern technology, medical breakthroughs in the identification of criminals remain a cumbersome task. The modern-day criminal investigation typically involves many different disciplines to solve a crime. The identification of a person's body is required when it is completely mutilated, disfigured, or beyond recognition -- which might be due to any barbaric crime, accident, war, fire, or any natural disaster. Dental identification is one of the most reliable methods, as teeth and dental structures may survive adverse conditions. The techniques involved in forensic odontology include: bite mark analysis, tooth prints, rugroscopy, cheiloscopy, dental DNA analysis, radiographs, and photographs.

## Introduction and background

History/past events of victim identification

The ancient dental identification began with the Agrippina and the Lollia Paulina case in the year 49 AD. It was determined that Lollia's teeth had some distinctive features which were used to identify her after her death [1]. 

Luntz presented a case about Warren, who was killed by the British and was recognized by Paul Evere by ivory denture work that he had done for Warren. This was the first case of identification by a dentist [2-3]. 

The late President of Pakistan, General Zia-ul-Haq, who died in a plane crash in 1988, was identified from his dentition. The first dental identification from a mass disaster was made for a fire in Charity Bazaar in Paris, here antemortem (AM) and postmortem (PM) dental records were compared for identification of the dead [4]. 

Forensic odontology successfully identified Tsunami victims in southeast Asia in 2004 [5]. The late Prime Minister of India, who was killed in a terrorist attack, was also identified by his dental records [6]. Our past is full of evidence that depicts how forensic odontology has been such an impactful tool in identification [7].

## Review

Teeth and DNA

The tooth serves as the most valuable source of DNA as it is cast in a sealed, calcified box protecting DNA from extreme environmental conditions [7]. When morphologically evaluated, a single tooth can provide valuable information regarding the individual to whom the teeth belongs to. Because of the resistant nature of the dental tissues to assault such as incineration, immersion, trauma, and mutilation, teeth present an excellent source of DNA [8]. Genomic DNA found in the nucleus of the cells is used for forensic application which can be taken from dentin enamel or pulp. MTDNA (MITOCHONDRIAL DNA) is used, where genomic DNA is not available. MTDNA is maternally inherited so that it can be used in the identification of siblings [9]. Schwartz et al.;, in 1991, isolated HMW PROTEIN from teeth under various adverse conditions such as different pH, humidity, temperature, and storage. It was seen that these adverse conditions did not affect the protein in any way, bolstering the fact that the tooth protects DNA [10]. DNA isolated can be amplified by the polymerase chain reaction (PCR) technique to increase the DNA sequence and use it for identification. Only 2%-5% of the genes of DNA codes for a protein are used, and the other 95%-98% is essentially junk or the noncoding part. Variation in DNA sequence called polymorphism can be used to correlate with an individual. Radicular pulp tissue is a good source of DNA [11]. Sometimes odontoblastic processes that extend to the dentinal tubules, cellular cementum, soft tissues in the accessory canals, periodontal ligament fibers can also be used for extracting DNA. Different techniques and approaches have been implemented to extract DNA from teeth. The horizontal section through the cervical route appears to be the preferred method because it allows the rotary instrument to get material from the inner dentine in root canals while preserving the coronal structure, which is vital for morphological identification [12-13]. The powerful way to exploit, manipulate, isolate, and analyze the DNA at molecular levels is the DNA fingerprinting [14]. The dental identification from a mass disaster in comparison to the AM and PM records [15]. Orthograde filling, carious tooth, noncarious tooth, and dental extraction are a few factors that were followed during dental identification in historical times [16]. Microanatomy plays a vital role in the extraction of DNA, which results in the identification of degraded human beings [17]. The adverse conditions of the degraded human remain, and the DNA extraction is carried out by adjusting the technique to remove enamel, cementum, and pulp [18]. The mtDNA displays a pattern of fragmentation across many tissue types, improving the ability to estimate DNA survival in forensic specimens [19].

The usefulness of tooth in sex determination

Certain dental indices such as incisor index, mandibular canine index, and crown index have been derived from the linear measurement of teeth that is sexual. These can be used to identify males and females. The mandibular canines have the most significant dimensional difference in males compared to females. Crown diameters and the combination of root length are also used for sex determination. Nonmetric features such as the digital accessory ridge, Carabellis straight of upper molars, and shoveling of upper central incisors, can also be sometimes used for sex determination. The digital accessory ridge in canines is more pronounced in males, compared to females. Females exhibit a lesser number of cusps in the mandibular first molar compared to females. The presence of bar bodies in the pulp of the tooth can also help in differentiating between males and females. Vemuri et al. did a study at 100, 200, and 400 degrees Celsius, which showed females had fibroblast with peripheral bar chromatin condensation that the males lacked. An Amelogenin or 'Amell' (a human enamel protein) has different patterns in males and females. The 'Amell' gene is located in the X chromosome and Y chromosome in males, whereas females have two ALEL genes on the X chromosome [20].

The use of tooth DNA in blood grouping

Every individual has a unique blood group. Besides the blood, the blood group antigens are also present in semen, sweat, amniotic fluid, and saliva. As the tooth pulp contains a lot of blood vessels, it is more certain that blood group antigens are bound to be present in the pulp [21]. Studies conducted by Ballal and David showed that the blood group from dentine and pulp correlated with the blood collected from the extraction socket [22]. The PM changes in the pulp are seen very late, and the pulp remains protected in the tooth cavity, thus making the pulp tissues one of the best agents for blood group recognition. Various studies done by absorption-elution or the absorption-inhibition methods are used for ABO blood grouping and Rh typing [21, 23-24]. Another study by Aswath et al. was conducted to concentrate on the sensitivity and specificity of the dental pulp in identifying the ABO blood group and Rh factor [25, 26]. A study conducted by Sasmita et al. compared the blood grouping obtained by the deciduous tooth of children to the children's parents. Significant positive results were obtained. Thereby establishing the correlation between the DNA of a child and a parent [27-28].

Kramer found that the detection of blood grouping from heart issues, such as dentine and enamel, was unreliable and inefficient. This could be because they were calcified [29].

The usefulness of dental DNA in age estimation

Age estimation can be an essential tool in identifying a person [30]. As dental maturity is not affected by nutritional and endocrine status, it can be used as a tool for identification [31]. The various methods in the age estimation process are anthropometric measurements, skeletal maturation, dental age estimation, a combination of dental development, and anthropometric measurements [32]. Various methods utilized in age determination are as follows.

All the primary teeth erupt from 6 to 30 months of age, and exfoliation of these teeth can be a way of estimating a child's age. Moorrees et al.’s landmark publications are still considered the most reliable set of data in these areas [33]. The stages of root resorption were used by Morris for age estimation. Later, Gordon et al. gave a system of classification based on the schematic diagram, which became accepted worldwide [34]. Factors used for age determination, include an appearance of a tooth gem, earliest detectable mineralization, degree of completion of a un-erupted tooth, rate of formation of enamel, formation of neonatal lines, attrition of the crown, and transparency of root dentine [35-36]. In dentine, the incremental lines of Vonebener and contour lines of Owen are present and are used to estimate the age of fetuses and death. The size of the pulp-chamber indicates the amount of secondary dentine formation. Moore used the pulp chamber diameter ratio with the crown diameter to estimate age [37-38]. If the third molars fully erupt, it indicates that the age of individual is above 17 years, and the examination of the X-rays of the root formation is not complete -- one can definitely infer that the person was probably less than 25 years of age [39-40]. Gustafson used six dental changes connected with age, namely attrition, apical migration of periodontal ligament, deposition of secondary dentin, cemental opposition, root resorption, and transparency of root dentine. The age was estimated using the following formula [41-43]:

Age = 11.43 + 4.56*x*, where *x* is the total score. It was found that an increase in the total score corresponds to an increase in age.

Gustafson in 1950 studied the changes occurring in an individual's teeth and estimated age with some accuracy [42]. As age advances, L-aspartic acid will change to D-aspartic acid [43].

Foti et al. studied age identification in living and dead children with the guidance of linear regression. The formula can be applied based on the total number of erupted teeth and the type of tooth germ detected during the clinical examination and also on the radiograph. Kvaal et al. highlighted that the intensity of the fluorescence estimates the color of the human dentin and cementum which plays a role in the "half technique" age identification formula. This equation helps in age determination until 20 years of age [41, 44].

 The mandibular canines have the most significant dimensional difference in males, which estimates to find they are 12 years of age [45-46]. Even in ancient times, the age identification of living adolescents was important. According to the records, in historical rooms, adults were judged to be fit for service with the eruption of their second molar [47-48].

The usefulness of teeth in race determination

A tooth can also be helpful in the estimation of the race of an individual. Caucasians have a high prevalence. Carabelli proves the simplification of the Fisher system. However, a high prevalence of shovel incisors and complex Fisher system is seen in Asians [45-47]. 

Assessments of socioeconomic status, personal habits, oral health status, and occupation

Low-income status individuals will have poorly maintained teeth, a high number of cavities, inadequate fillings, and prosthetic replacements by cheap materials. On the other hand, the high-income individual will have well-treated, esthetically pleasing teeth. Orthodontic appliances may point to a person of high-income status. Open bite, cross bit, and protruded incisors may indicate inappropriate habits, such as thumb sucking in children. Personal habits such as smoking and tobacco chewing can also help to identify individual defects on dental heart issues. Erosive changes due to bulimia and anorexia can be determined by looking at the tooth. It is possible to find dental defects mostly seen in the upper and lower incisal edges of incisors. For example, glass blowers, shoemakers, and musicians who play wind instruments will have an attrited incisal edge. The high cavity index can determine the diet of individuals, i.e., if he or she takes in more carbohydrates or sugar, they will have more cavities [49].

Teeth in bite marks

The human occlusion profile is different for everyone. There is a small hypervariation that can be used to create a database. But, the only drawback is that this hypervariation is not constant throughout life as compared to DNA, which is constant. Bite marks were used in ancient history for the identification of an individual. William, the conqueror, validated the royal documents by biting into the wax seal with his characteristic bite marks. In Britain and Europe, debtors' who worked as servants were verified by their bite marks in lieu of their signatures. 

There are several important steps that a person needs to follow to identify and report a bite mark. This includes identifying bite marks, documenting it, preserving it, dental profiling of the evidence and suspect, DNA profiling through available salivary swab, and finally reporting the crucial evidence. 

The American Board of Forensic Odontology (ABFO) no. 2 standard reference scale has been recognized by the forensic science community as a geometric reference scale. It is a photo macro graphic ruler designed to optimize the ability to reconstruct a bite mark, skin trauma, a scene, or an object from an image.

Different methods applied to compare the suspect dentition and bite marks injury physically are fingerprints, dusting powder, 3D laser scanning of dental casts, confocal scanning, and electron microscope.

Analyzing bite marks with image perception technology was proposed by van der Waals et al. He stated that it is possible to color areas with equal intensity values and depict a 2D image as a pseudo-3D surface object. The ABFO and the British association of forensic odontology have published guidelines that suggest that evidence should be collected from both victims and suspects. Deviations from these recommendations may be questioned. An overestimation of bite marks should always be avoided. One should never forget that bite marks are not as helpful as DNA in an identification [50].

Comparative dental identification

The nuances and complexities of this process should be dealt with carefully. The AM records such as case history, radiographs, photographs, full mouth impression, presence or absence of pathologies, periodontal health can be used in the positive identification of disease when compared with the PM finding. Sometimes when the AM dental records are unavailable and other methods of identifications are not possible, PM dental profiling can be done [13]. An Individual's teeth and the condition of the oral cavity can be checked to assess the socioeconomic status, sex and ancestry background, dietary habits, etc. [49].

The need of the hour for forensic odontologists

Forensic odontologists play an active role in the victim identification process. This multidisciplinary approach is important in the following sections for the best outcome of forensic analysis.

Border control: Active cross-border terrorism at the national boundaries with neighboring countries is causing many soldiers to become martyrs. Their body identification is becoming difficult because of burns and mutilations. In this case, forensic odontology can help in the identification of individuals by comparing the dental records collected before the commencements of his or her service.

Human trafficking: It is the second-largest criminal activity in the world. Dental professionals can contribute to the identification of the trafficked person and help the police in an investigation against the criminal organization.

Identification of nameless cadavers: Forensic odontology can provide evidence to families who may be used in court to show that the body is available to the family for the funeral.

Disaster victim identification (DVI): Forensic odontologists can play an important role in the identification of mass disasters across the world [*].

## Conclusions

Teeth have proven to be a very helpful adjunct in forensic dentistry. They provide a huge amount of information in the field of DVI, missing persons, unidentified persons, child abuse, domestic violence, and age estimation. A great amount of research is being done currently that could yield extremely beneficial results in the future.
